# Pressure pain thresholds in a real-world chiropractic setting: topography, changes after treatment, and clinical relevance?

**DOI:** 10.1186/s12998-022-00436-2

**Published:** 2022-05-12

**Authors:** Casper G. Nim, Sasha L. Aspinall, Rasmus Weibel, Martin G. Steenfelt, Søren O’Neill

**Affiliations:** 1 Medical Research Unit, Spine Centre of Southern Denmark, University Hospital of Southern Denmark, Middelfart, Denmark; 2 Department of Regional Health Research, University of Southern Denmark, Odense, Denmark; 3 College of Science, Health, Engineering and Education, Murdoch University, Perth, Australia

**Keywords:** Spinal manipulation, Pressure pain threshold, Topograhic mapping, Pragmatic research

## Abstract

**Background:**

Changes in pain sensitivity are a commonly suggested mechanism for the clinical effect of spinal manipulative therapy (SMT). Most research has examined pressure pain thresholds (PPT) and has primarily been conducted in controlled experimental setups and on asymptomatic populations. Many important factors are likely to differ between research and clinical settings, which may affect PPT changes following SMT. Therefore, we planned to investigate PPT before and after clinical chiropractic care and investigate relationships with various potentially clinically-relevant factors.

**Methods:**

We recruited participants from four Danish chiropractic clinics between May and August 2021. A total of 129 participants (72% of the invited) were included. We measured PPT at eight pre-determined test sites (six spinal and two extra-spinal) immediately before (*pre-session*) and immediately after (*post-session*) the chiropractic consultation.

We used regression analyses to investigate PPT changes, including the following factors: (i) vertebral distance to the nearest SMT site, (ii) rapid clinical response, (iii) baseline PPT, (iv) number of SMTs performed, (v) at the region of clinical pain compared to other regions, and (vi) if other non-SMT treatment was provided. We also performed topographic mapping of pre-session PPTs.

**Results:**

After the consultation, there was a non-significant mean increase in PPT of 0.14 kg (95% CIs = − 0.01 to 0.29 kg). No significant associations were found with the distance between the PPT test site and nearest SMT site, the clinical response of participants to treatment, the pre-session PPT, the total number of SMTs performed, or the region/s of clinical pain. A small increase was observed if myofascial treatment was also provided. Topographic mapping found greater pre-session PPTs in a caudal direction, not affected by the region/s of clinical pain.

**Conclusions:**

This study of real-world chiropractic patients failed to demonstrate a substantial local or generalized increase in PPT following a clinical encounter that included SMT. This runs counter to prior laboratory research and questions the generalizability of highly experimental setups investigating the effect of SMT on PPT to clinical practice.

**Supplementary Information:**

The online version contains supplementary material available at 10.1186/s12998-022-00436-2.

## Background

Quantitative sensory tests (QST), in which controlled painful stimuli are used to assess various aspects of pain sensitivity, have been widely used to explore the neurophysiology of pain, especially chronic pain, and the effects of various therapeutic interventions [1, 2]. Spinal manipulative therapy (SMT) is one such intervention that is widely used in the conservative management of spinal pain. Spinal manipulative therapy is most often delivered in the form of a high-velocity low-amplitude (HVLA) thrust targeting a spinal joint, and most of the research on SMT uses HVLA SMT [3].

The most commonly used QST procedure in manual therapy research is pressure pain threshold (PPT). Recent systematic reviews have concluded that PPT increases (indicating decreased pressure pain sensitivity) at least short-term after SMT in symptomatic [4] and asymptomatic populations [5]. However, there are various weaknesses in this body of research, including concerns about shortcomings in research methodology and a lack of sham-controlled studies [4].

Additionally, the clinical relevance of these changes is unclear. Many of the included studies in the systematic reviews have assumed a relationship between changes in pain sensitivity as measured using QST and clinical outcomes after SMT, but few have investigated it specifically [4–7]. The underlying theories appear to be that SMT affects pain sensitivity either by a direct, local reflex mechanism, by triggering descending pain inhibition, or by alleviating a painful clinical condition and thereby indirectly normalizing pain sensitivity. We have previously reported that PPT increased more after SMT targeting the most painful lumbar vertebra than the stiffest vertebra, although there were no differences in clinical outcome [8]. In a secondary analysis, PPT increase was associated with clinical improvements following the SMT regardless of the SMT site [9]. In contrast, a recent randomized controlled trial found that changes in PPT did not consistently relate to rapid improvement in low back pain after SMT or sham SMT [10]. The research findings in this area are not clear or consistent.

Another topic of interest is the topographical mapping of QST, which provides data on the spatial heterogeneity of QST in various populations [11]. Alterations in QST, and differences between test sites, have potentially valuable implications for our understanding, prognosis, and management of painful conditions [2, 12–14]. This knowledge can also inform the selection of QST test sites in future research, which in turn has the potential to help practitioners of SMT in selecting an appropriate site for SMT [15].

Another relevant concern, which appears to have received little attention, is the generalizability of such laboratory QST research to the clinical settings where manual care for painful conditions is typically delivered to patients. There are many differences between typical experimental research settings and clinical settings (e.g., the inclusion of participants with pain and lack of focus on contextual factors such as the therapeutic alliance).

The importance of contextual effects in manual therapy has received increasing attention recently [16, 17] and is pertinent when considering pain-related outcomes. Contextual effects refer broadly to aspects of a patient encounter outside of the “specific” active effects of any interventions delivered. Contextual factors include the physical environment, the therapeutic relationship, language and communication, beliefs and expectations of the patient and the clinician, rituals, other general features of the interventions delivered, and more [17]. This also incorporates the more well-known phenomena of placebo and nocebo. It has been suggested that contextual factors may account for a significant “non-specific” portion of a patient’s response to care, including manual therapies [17].

Most QST studies are performed in highly controlled research environments where prescriptive procedures are followed. There may be an emphasis on potential risks related to testing procedures as necessitated by the informed consent process. Conversely, there is little if any emphasis on relationship-building and shared therapeutic goals in interventional studies. A substantial body of research on QST and manual therapy has also been done on asymptomatic populations, which likely impacts beliefs and expectations and physiological effects of SMT [5–7]. In real-world clinical settings, however, there is more emphasis on building relationships. The environment is likely to be more relaxed/welcoming, and the motivation for patients to seek treatment is very different from participation in a research project. How interventions are delivered differs too; experimental studies often deliver highly prescriptive and limited interventions, sometimes to pre-determined anatomical locations. In clinical practice, by contrast, interventions are delivered more pragmatically, tailored to the individual patient, and may involve multiple manual interventions, advice, education, reassurance, and exercises.

Thus, many important factors are likely to be very different in research versus real-world settings, and these differences may well affect the outcome of QST tests before and after SMT. Therefore, we planned to investigate PPT before and after real-world chiropractic care in patients attending their regular chiropractor and investigate relationships with various potentially clinically-relevant outcomes.

## Objectives

The primary aim (A) of this study was to assess changes in PPT at the different vertebrae following SMT and to explore the modifying effect of six additional factors:

(A.I) The vertebrae receiving SMT (i.e., the proximity of the PPT measure to the SMT site)
(A.II) Rapid clinical responsiveness
(A.III) The baseline PPT value (i.e., do participants with lower PPTs display more pronounced changes in PPT following the SMT)
(A.IV) Number of SMTs performed
(A.V) The region of clinical pain (i.e., do areas closer to the painful region tend to change more or less)
(A.VI) Other non-SMT treatments provided

Our second aim (B) was to describe the spinal and extra-spinal topographic mapping of PPTs in primary care chiropractic patients by investigating differences:

(B.I) Between vertebrae
(B.II) Between vertebrae in reference to the region of clinical pain

## Methods

### Design and setting

We used a pre-post treatment study design conducted in primary chiropractic care practices in the regions of Southern and Central Denmark. The study was approved by the Regional Committee on Health Research Ethics for Southern Denmark (S-20210035). The manuscript was prepared in reference to the STROBE format for observational studies [18].

### Participants

Inclusion criteria were: (i) 18 years or older, (ii) able to read and write Danish, (iii) primary complaint of spinal pain (in any region) with or without radiculopathy. Exclusion criteria were (i) competing diseases that could affect the central nervous system (psychological or somatic). All participants with complete data (pre and post-consultation) and who actually received SMT were analyzed.

We used convenience sampling, where potentially eligible patients were invited to participate by the secretary of the chiropractic clinics or the chiropractor when booking a new or follow-up appointment. Patients enrolled as participants came in 30 min before their chiropractic consultation. Formal information about the study procedure, confirmation of inclusion criteria, written information was provided, and signed consent was obtained before the initial assessment by the research assistants MGS and RW at the clinic.

### Procedure

The study assessed PPTs at two different times, immediately before the chiropractic consultation (*pre-session*) and immediately after (*post-session*). We did not limit inclusion to any specific visit (e.g., initial examination and follow-up treatment). Each QST session took approximately 15–25 min pre consultation and 5–10 min post-consultation.

### Pressure pain threshold assessment

#### Test sites

The locations of eight anatomical test sites (six spinal and two extra-spinal) were marked with a black felt-tip pen to ensure the markings were visible following the consultation. During the pre-session, participants lay comfortably in the prone position on an examination plinth suitably undressed for the QST procedures. The test sites were located by palpation following a strict protocol as below [19–22].

*C3***:** By locating the inferior edge of the occipital bone and palpating bony prominences in the inferior direction until the second bony prominence was located and marked as the spinous process of C3.

*C7***:** By passively flexing and extending the participant’s head using the head support, attempting to locate the spinous process of C6 by palpating for anterior movement when extending the neck. The bony prominence just below was marked as the spinous process of C7.

*T3 and T7:* By locating and counting spinous processes in the inferior direction from C7 until the spinous processes of T3 and T7 were reached and marked. T7 was checked by depicting a straight horizontal line from the inferior angle of the scapula onto the thoracic spine.

*L1 and L5***:** By depicting a straight horizontal line between the two iliac crests and locating the spinous process at the closest superior proximity to that line (L4), we then palpated in the superior direction to L1 and the inferior direction to L5. If locating L1 proved difficult, we attempted to confirm our location by palpating costae 12 and identifying the spinous process of T12, then palpating inferiorly to L1.

*Infraspinatus:* By locating the midpoint of the scapula’s spine and measuring 2 cm directly inferior, we marked the belly of the infraspinatus muscle.

*Tibialis anterior:* By locating the inferior border of the patella, we measured 8 cm inferior and moved 2 cm laterally from the tibia, here marking the belly of the anterior tibialis muscle (this was done with the participant in a sitting position).

The eight sites were selected to cover the spinal junctions and the mid-sections, allowing us to assess as much of the spine as possible.

Pressure pain threshold was tested 2 cm lateral to the identified spinous process at the spinal sites in the paraspinal muscle belly. The test side was either on the side indicated by participants as the most painful side of their clinical complaint or on the dominant hand side if participants could not identify a more painful side.

We used an a-priori, computer-generated randomized test order for each subject, including all eight test sites. Two research assistants performed the PPT procedure following a detailed, scripted procedure which included exact wording of verbal instructions and the PPT procedure. The research assistants (MGS and RW) underwent extensive training independently and were supervised by either CGN or SON on multiple occasions before data collection began [23].

#### Pressure pain threshold

**Fig. 1:**
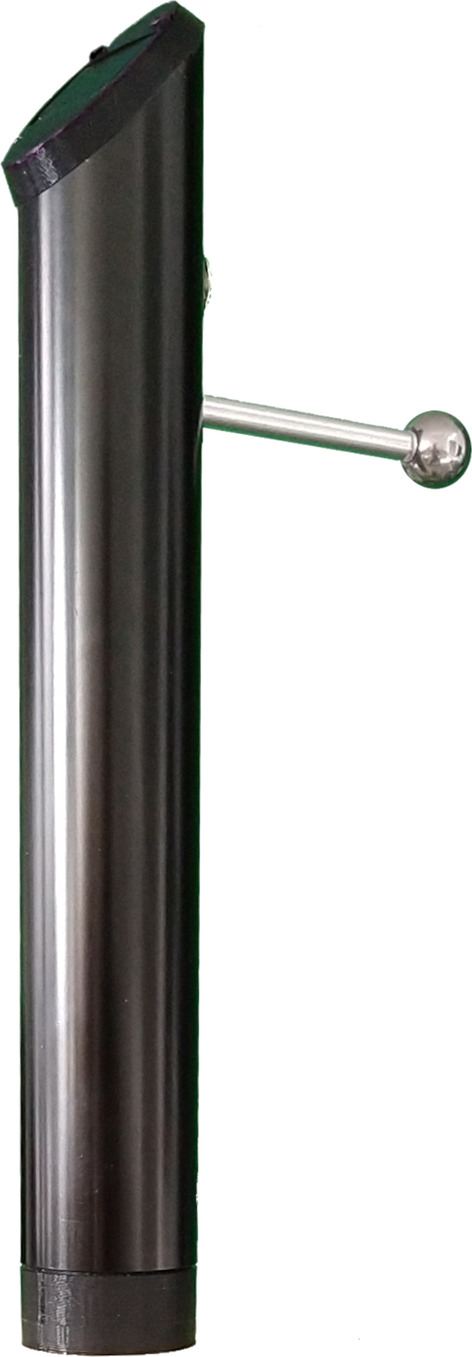
The custom-made algometer used to test pressure pain thresholds in chiropractic patients

To assess PPT, we used a custom-made pressure algometer (built on Arduino Nano v.3, HX711 weighing cell amplifier and A2D converter) with a spherical probe-head with a diameter of 1.5 cm (Fig. 1) [24]. A spherical probe head was chosen to help ensure a measure of *deep* PPT and minimize mechanical stimuli/strain of the skin, as previously recommended [25]. The PPT was measured by applying a gradually increasing pressure of approximately 0.5 kg per second (guided by visual feedback through a speedometer of rate of change on the algometer’s display) perpendicular to the skin until the participant indicated the pressure was painful by pressing an indicator button. Each PPT was performed once per test site with 10-s intervals. If no pain was elicited by 10 kg (kg) of pressure, this was recorded as the PPT. For safety concerns, we limited the pressure at C3 to 5 kg.

Before the PPT assessment started, each participant was provided with detailed instructions. Two test procedures were performed at the non-dominant forearm and one at the posterior thigh to familiarize participants with the procedure. The same research assistant performed both tests (pre and post) on the same participant to limit inter-tester variation.

The PPT value was not shown to the research assistants on the algometer until after the indicator button was pressed to limit response-shift bias. The non-testing research assistant recorded the PPT value in a separate spreadsheet (Microsoft Office Excel for Windows 10) blind from the testing research assistant. Finally, to prevent any order effect of the test sites, the sites were tested in a computer-generated random order.

## Variables of interest

### Pressure pain threshold

The PPT value at each test point is presented as a continuous pressure in kg [0–10]. As we used a spherical probe with a diameter of 1.5 cm, we cannot assume (i) how much of the sphere touches the participant’s skin, (ii) how much pressure is exerted across the sphere, and (iii) the different mechanical properties of their skin/muscle. Therefore, estimating the pressure as force over the area is not a simple task, and we have opted to report it as a force while maintaining the commonly used term “pressure pain threshold.”

We calculated “change in PPT” values for each participant and test site by subtracting the post-session from the pre-session. Thus, a negative change in PPT value indicates a decrease in PPT, and a positive change in PPT value indicates an increase.

### Patient-reported variables

Participants answered questionnaires at the pre-session about their clinical status. All variables were recorded using SurveyExact (Ramboll, Denmark) and answered directly in the examination room on an iPad (Apple, USA). Participants also assessed their rapid response status at the post-session before PPT was re-measured.

Demographic data:

- Sex [male/female/other]
- Age [years]

Spine pain status:

- Region of pain [lumbar, thoracic, cervical, or any combination]
- Current spine pain [0–10, 0 would indicate no pain, and 10 the worst possible pain imaginable]
- Duration of the spine pain [0–1 weeks, 1–2 weeks, 2–4 weeks, 4–12 weeks, > 12 weeks]

Current chiropractic visit:

- Number of visits [1, 2, 3, 4, or 5 times, more than 5 times, or “I come in regularly after a certain period” (i.e., maintenance care)]
- Rapid response [7-point Likert scale, anchored with “much worse” to “much better” with “no change” in the middle (answered at post-session)]

### Clinician-reported variables

The treating chiropractor recorded the following, on a paper chart, after treating the participant:

- Where in the spine SMT was performed
- Number of SMTs provided
- Free text field to elaborate on the SMT
- Other non-SMT interventions provided

Following completion, the chart was placed within a sealed opaque envelope and returned to the Spine Centre of Southern Denmark for safekeeping until completion of data collection. The chart was designed (CGN and SON) to collect relevant information about the SMT provided and pilot-tested by the chiropractors enrolled before use. Following this procedure, we made no amendments to the chart (Additional file 1).

#### Data extraction

When data collection was concluded, two authors (MGS and RW) independently extracted the data from the chiropractors’ charts and compared their recordings. Any disagreement would be resolved by a referee (CGN). However, a referee was not required.

### Statistical analysis plan

All data cleaning, visualizations, and analyses were conducted using R vers. 4.1 with R-studio vers. 1.4 [26] for Zorin OS 16 (Linux, Ubuntu 20.04) using the *Tidyverse language* [27], with add-on packages (listed accordingly).

### Sample size calculation

We expected to see an outcome change in PPT with an effect size of 0.25 [4]. Provided an alpha value of 0.05 and a beta-error of 0.80, a pre-hoc sample size calculation indicated that we needed 129 participants. However, to account for loss of follow-up or erroneous data, we aimed to include a maximum of 140 participants but would stop at 129 participants with usable and complete data independent of their SMT status – as this factor was unknown.

### Descriptive data

First, participants’ demographic and clinical data were tabulated with proportions and means as appropriate. Second, PPT data across the eight test sites were tabulated with means, medians, SDs, and interquartile ranges (IQRs) as we expected this variable not to be normally distributed. Third, where the SMT was applied was illustrated using a bar chart as the proportions at each vertebra by their region of pain. Results are reported using the *gtsummary package for R* [28].

### Research aims

Linear mixed models were computed and reported using the *LM4 and LMERtest packages for R* [29, 30]. All relevant model assumptions were checked visually (linearity, homogeneity of variance, normality of residuals, and random effects) using the *EasyStats performance package for R* [31]. Results are extracted and reported using the *EasyStats model-based and report packages for R* [32].

### Aim A—changes in pressure pain threshold following chiropractic treatment

Changes in PPT pre/post-treatment were presented as mean changes and 95% confidence intervals (CI) for all test sites combined and for each test site. Each sub-component of *Aim A* was described in Table 1. All data-frames used for modeling were reduced, reporting as few degrees of freedom as possible, not artificially increasing the sample size [33]. For multilevel data, we applied linear mixed models.

**Table 1:** A description of the applied regression models for Aim A

Aim A objective	Method	Fixed dependent effect	Fixed independent effect	Random intercept	Reported as
Changes in PPT over time	Linear mixed regression	PPT	Model 1: Time Model 2: Interaction between time and test site	Subject	Mean change value with 95% CI, both dependent and independent of the test site
(I) Distance from test site to SMT site	Linear mixed regression	PPT change	Distance between the tested vertebrae and the closest SMT site (e.g., PPT test at C7 and SMT at C1 = distance of 6)	Subject + PPT at baseline + region of pain	A Beta coefficient with a 95% CI. A scatter plot with a best fitting regression line
(II) Rapid responder status	Linear regression	PPT change	Responder status (e.g., much better)	–	Between group mean differences for each rapid responder status change values with 95% CI
(III) The baseline PPT value	Linear regression	PPT change	The baseline PPT value (e.g., 5.5 kg)	–	A Beta coefficient with a 95% CI
(IV) Number of SMTs performed	Linear regression	PPT change	Number of SMTs performed (e.g., 3 SMTs)	–	A Beta coefficient with a 95% CI
(V) Region of pain compared to the adjacent or distant region*	Linear mixed regression	PPT change	Pain region (i.e., change in PPT in the pain region (e.g., cervical) compared to change in PPT in the i) adjacent region (e.g., thoracic) or ii) distant region (e.g., lumbar))	Subject	Between group mean differences for the pain region and adjacent/distant region with 95% CI
(VI) Other non-SMT treatment provided	Linear regression		Non SMT-treatment provided. Categorized as myofascial (compression technique or dryneedling), muscle energy technique, massage, other (Laser or free text field), and none	–	Between group mean differences of the different non-SMT options with 95% CI

^*^For objective V, we used the mean value for each region (e.g., mean of PPT from L1 and L5), and for those with multiple pain sites, the “pain region” was determined as any point of pain (e.g., for those with lumbar and thoracic pain, both the lumbar and the thoracic PPTs would be flagged as “the pain region” with the cervical region being the “adjacent”). CI = confidence interval, PPT = Pressure pain threshold, SMT = spinal manipulation

### Aim B—topographic mapping

Using linear regression with pre-session PPT as the dependent variable and the independent variables were test sites (B.I) and the interaction between test sites and region of pain (B.II), we present a heat map with mean differences and 95% CI. We determined differences in PPT between the test sites and how this interacted with the region of clinical pain. We summarized the PPT values as mean values across test regions (i.e., cervical, thoracic, lumbar, non-spinal) for ease of interpretation. Results were tabulated with mean differences and 95% CI and presented as a line plot.

## Results

A total of 17 chiropractic clinics were invited to participate, and from those, we included four clinics. Seven clinics did not respond to repeated inquiries, and the remaining six could not accommodate it into their daily planning (e.g., they did not have a free examination room).

**Fig. 2:**
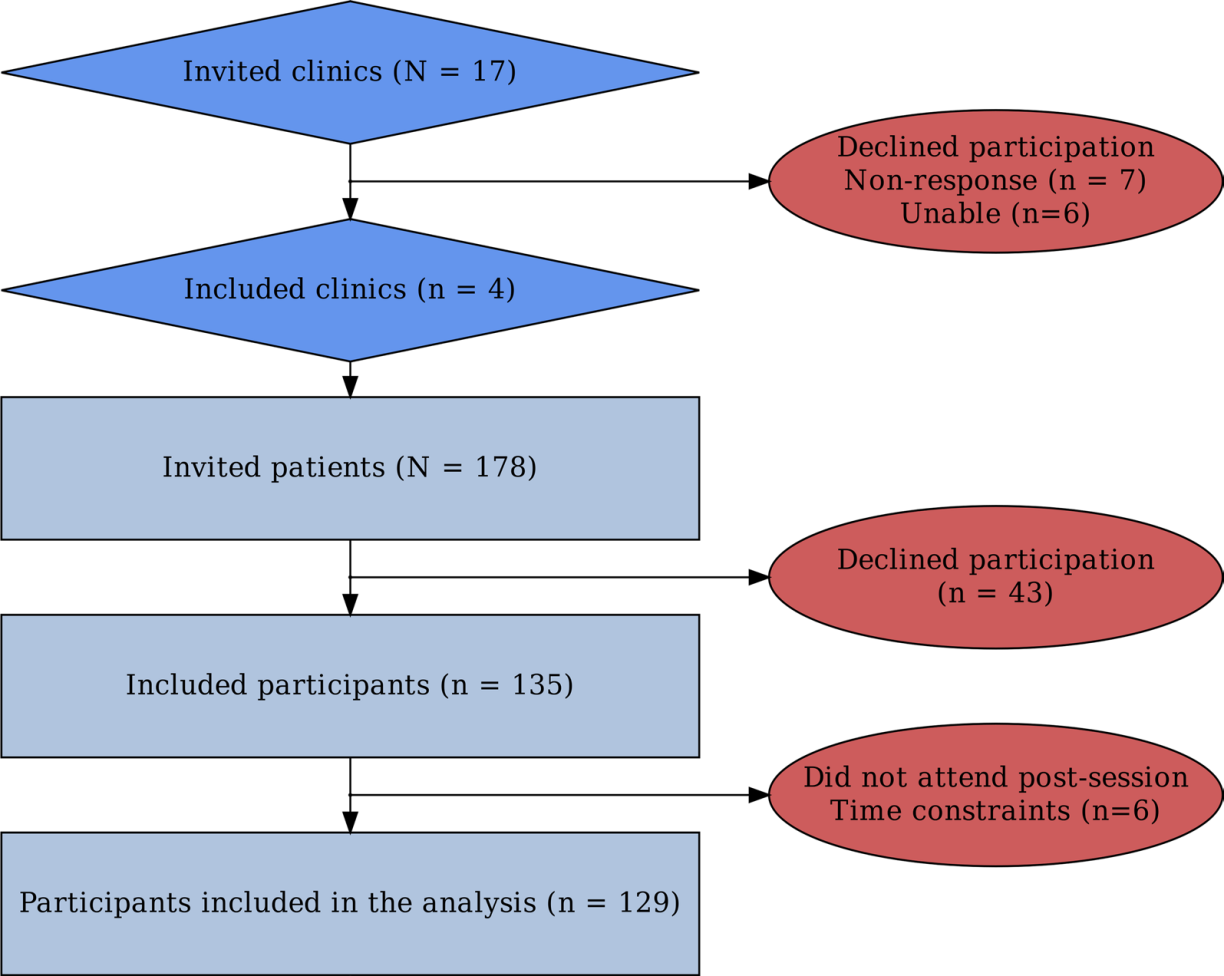
Flowchart of the included clinics and participants

The four participating clinics invited 178 patients, and 43 declined to participate, primarily due to lack of time or willingness. No participants were excluded as per exclusion criteria. Eleven chiropractors enrolled 135 participants between May 14th, 2021, and August 30th, 2021. A total of six participants did not participate in the post-session due to lack of time. Thus, leaving the desired sample of 129 with complete data (72%) (Fig. 2).

The enrolling and treating chiropractors had on average 14 years of experience (range 3–27 years) and were primarily educated from the University of Southern Denmark (7/11).

### Descriptive data

The included participants were primarily low back pain patients with acute episodes of less than 12 weeks of pain. They were principally seen at the beginning of their treatment (visits number 1 to 4). However, the sample also included participants with long pain duration and multiple pain sites. Approximately 35% only received SMT and if a second intervention was applied this was primarily myofascial treatment (Table 2).

**Table 2:** A descriptive table of 129 Danish chiropractic patients

Characteristic	N = 129^a^
Sex, male	67 (52%)
Age, years	50 (16)
Pain duration, weeks	
0–1	24 (19%)
1–2	28 (22%)
2–4	16 (12%)
4–12	13 (10%)
12 or more	48 (37%)
Back pain intensity [0–10]	5 (2)
Number of visits	
1	35 (27%)
2–4	47 (36%)
5 or more	25 (19%)
Maintenance care	22 (17%)
Region of pain	
Cervical	25 (19%)
Thoracic	8 (6%)
Lumbar	65 (50%)
Cervical + Thoracic	9 (7%)
Cervical + Lumbar	9 (7%)
Thoracic + Lumbar	6 (5%)
Cervical + Thoracic + Lumbar	7 (5%)
Non-SMT treatment provided	
Myofascial	55 (43%)
Massage	21 (16%)
Muscle energy technique	2 (2%)
Multiple	5 (4%)
Other	1 (1%)
None	45 (35%)

^a^n (%); Mean (SD)

**Fig. 3:**
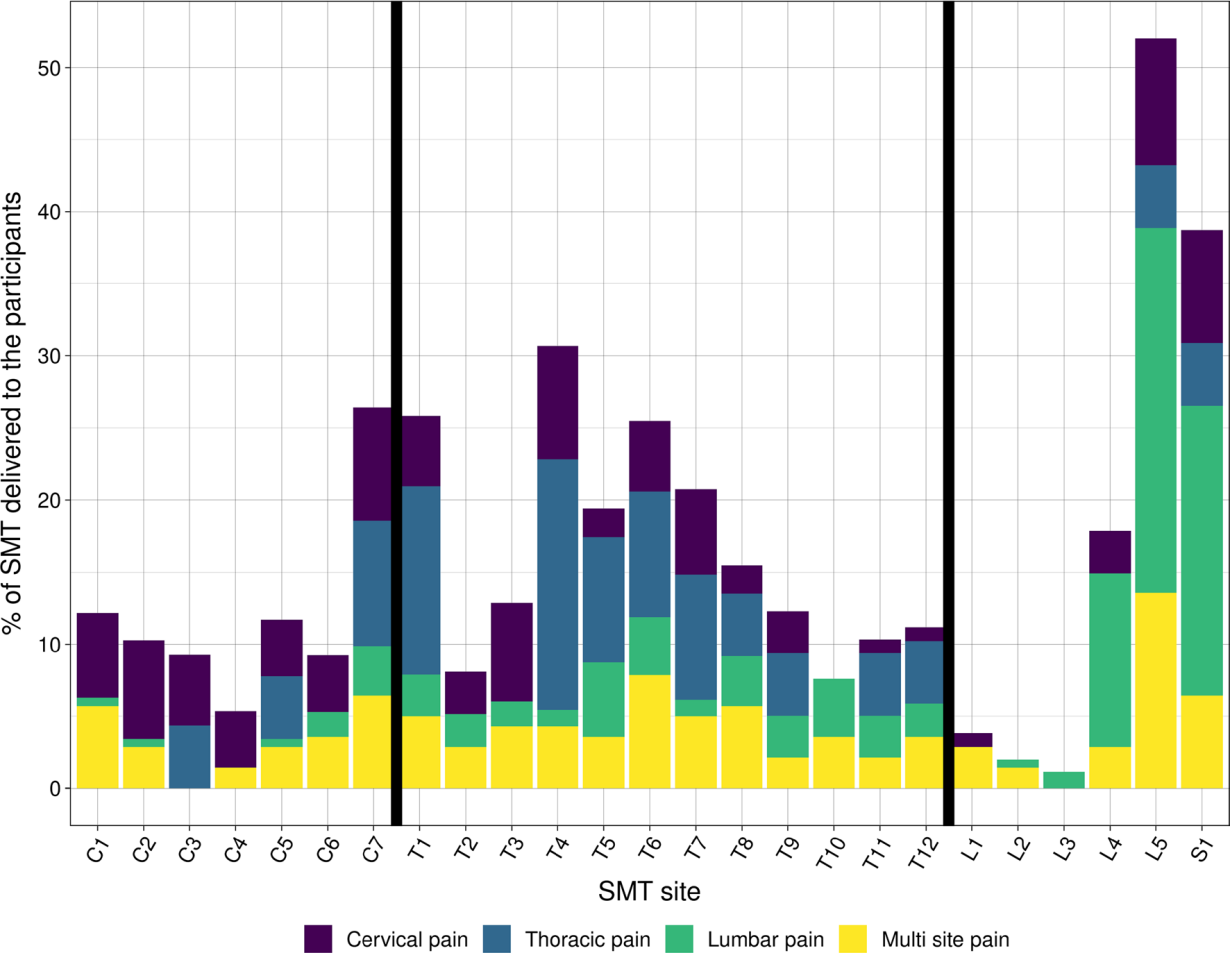
The proportion of SMTs targeting each vertebra subgrouped by participant’s region of pain. SMT = Spinal manipulation

Participants with lumbar pain had a higher proportion of SMTs targeting the lumbar vertebrae with limited SMTs targeting the cervical. Likewise, thoracic pain (n = 7) also had the highest proportion of SMTs targeting the thoracic vertebrae. Participants with cervical and multiple pain sites SMTs were distributed across all regions. Most SMTs were provided at the cervicothoracic junction, mid-thoracic spine, and lumbosacral junction (Fig. 3).

### Aim A—changes in pressure pain threshold following chiropractic treatment

The mean change in PPT across all test sites, independent of any other factors, was 0.14 kg (95% CI = − 0.01 to 0.29 kg, *p* value = 0.07). Thus, the mean change observed was close to 0 kg. The change in PPT was also not statistically significant when we analyzed each test site separately. An overview of the mean changes at each test site can be found in Additional file 2.

**Fig. 4:**
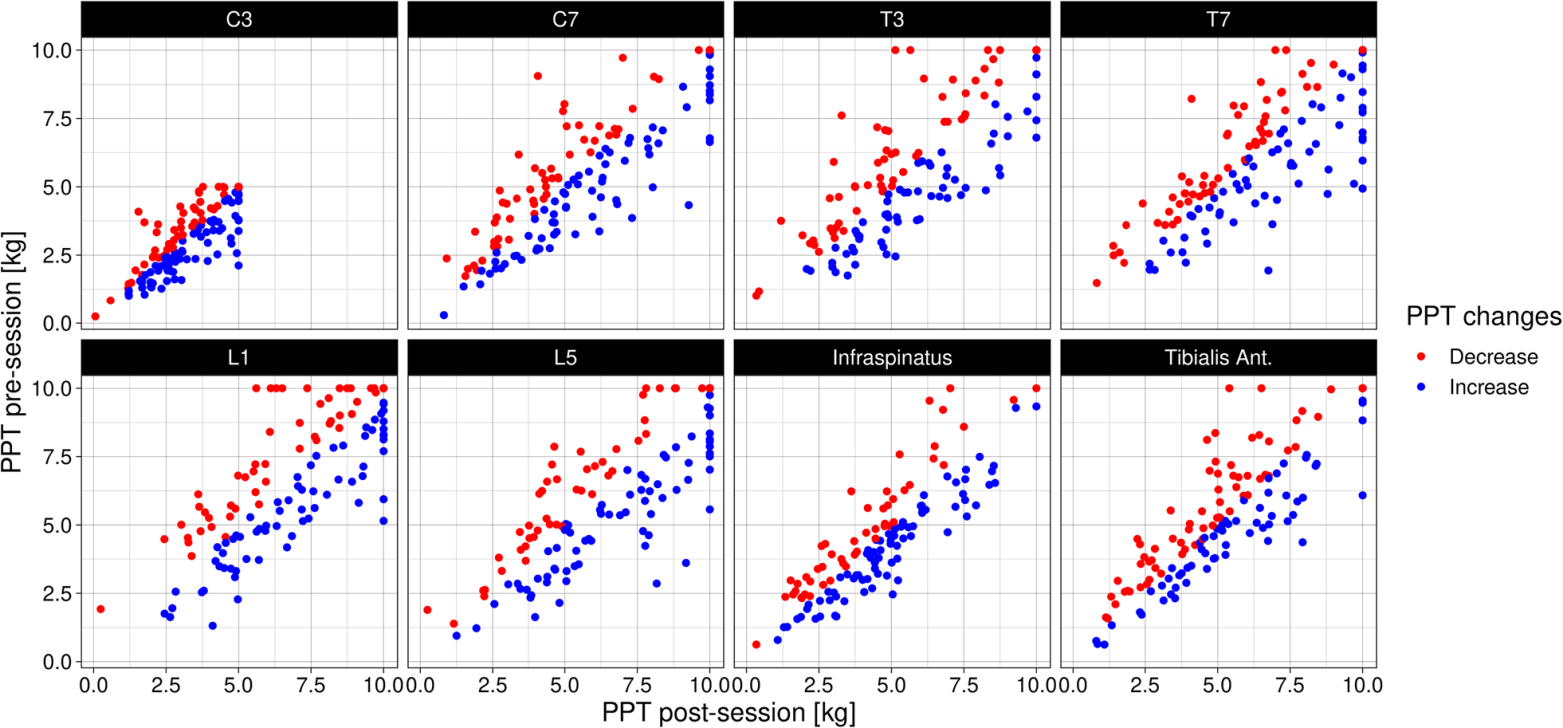
Pressure point threshold values pre and post a spinal manipulation session at different test sites. A red point indicates an overall decrease of PPT, while a blue point indicates an overall increase. PPT = Pressure pain threshold

**Fig. 5:**
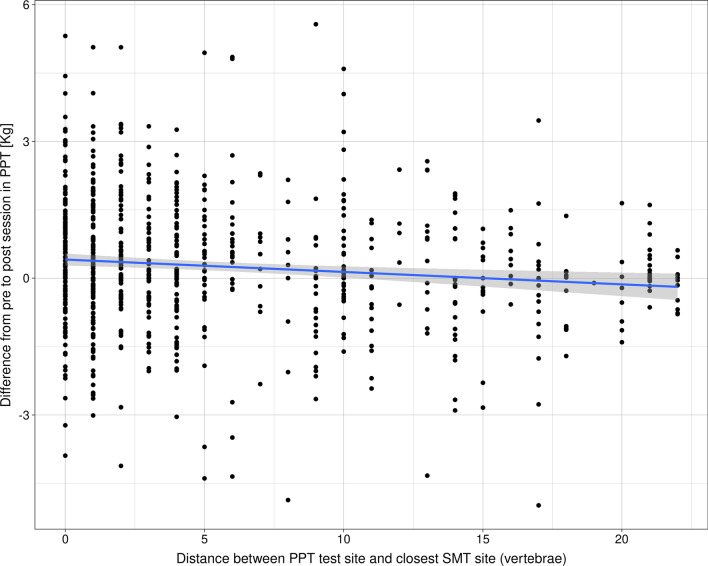
The difference in pressure pain threshold at different test sites in relation to the distance from the site of spinal manipulation. The distance is measured in vertebrae (e.g., distance from L1 to L5 = 5). The blue line indicates the best-fitting regression line with the 95% confidence interval. PPT = Pressure pain threshold, SMT = Spinal manipulative therapy

However, when looking closer at the individual data points, the mean group change of ~ 0 reflects individual participants with substantial increases in PPT and others with substantial decreases. A ceiling effect may have also affected the mean change (Fig. 4), though it was only the minority who were close to the upper limit at the pre-session (~ 11%).

(I) Distance from test site to SMT site
  A small association between change in PPT and distance to the closest SMT site was observed, with a Beta coefficient of − 0.03 Kg (95% CI = − 0.05 to − 0.01 Kg, *p* value <0.01). The model’s intercept was 0.48 Kg (95% CI = 0.13 to 0.83 Kg), which means that the predicted mean PPT increase at the SMT site was greater than the predicted mean change at test sites further away. However, with a coefficient of only − 0.03 kg, this effect was essentially negligible. In other words, any effect of SMT on PPT does not appear to be particularly related to the vertebra or even regional.
  When examining Figure 5, we also observed that when there was a distance of less than around 15 vertebrae between the test site and nearest SMT, PPT tended to increase after SMT. The opposite was observed for longer distances.
(II) Rapid responder status
  The majority of participants reported some level of improvement in symptoms with treatment (slightly worse = 6, no change = 28, slightly better = 37, better = 38, and much better = 20). Therefore, we dichotomized the outcome into a rapid responder status based on *unchanged* (slightly worse, no change, or slightly better) or *improved* (better or much better). There was no significant association between changes in PPT and rapid responder status (Beta coefficient of 0.07 Kg, 95% CI = − 0.21 to 0.34 kg, *p* value = 0.63). We present our a-priori specified analysis in Additional file 3 for completeness, but the conclusion is similar: no difference in PPT change based on short-term symptomatic response to care.
(III) The baseline PPT value
  There was a small, non-significant association between the baseline PPT value and the change in PPT, with lower PPT values at baseline being associated with a smaller increase in PPT at the post-session (Beta coefficient of − 0.04 Kg, 95% CI = − 0.11 to 0.04 kg, *p* value = 0.34).
(IV) Number of SMTs performed
  The median number of SMT treatments provided per patient was 3 (IQR of 3, range of 1–12) and no significant relationship was observed between number of SMT treatments provided and changes in PPT (Beta coefficient of 0.04 kg, 95% CI = − 0.03 to 0.11 kg, *p* value = 0.25).
(V) Region of pain compared to the adjacent or distant region
  Pressure pain thresholds tended to increase more in the clinical pain region than in adjacent or distant regions. However, the CI’s cross zero for both comparisons, meaning the differences are non-significant (Table 3).
(VI) Other non-SMT treatment provided
  There was a small and statistically significant difference between those who received myofascial technique treatment in addition to SMT to those who only received SMT (Beta coefficient of 0.53 Kg, 95% CI = 0.08 to 0.98 kg, *p* value = <0.01). All other comparisons were not statistically significant (Additional file 4).

**Table 3:** The mean change in pressure pain threshold at the region of pain compared to other spinal regions

Parameter	Difference in change in PPT compared to painful region (95% CI)
Adjacent to the pain region	− 0.08 (− 0.35–0.19)
Distant to the pain region	− 0.20 (− 0.51–0.11)

N = 129, CI = Confidence interval. PPT = pressure pain threshold

### Post-hoc analysis

**Fig. 6:**
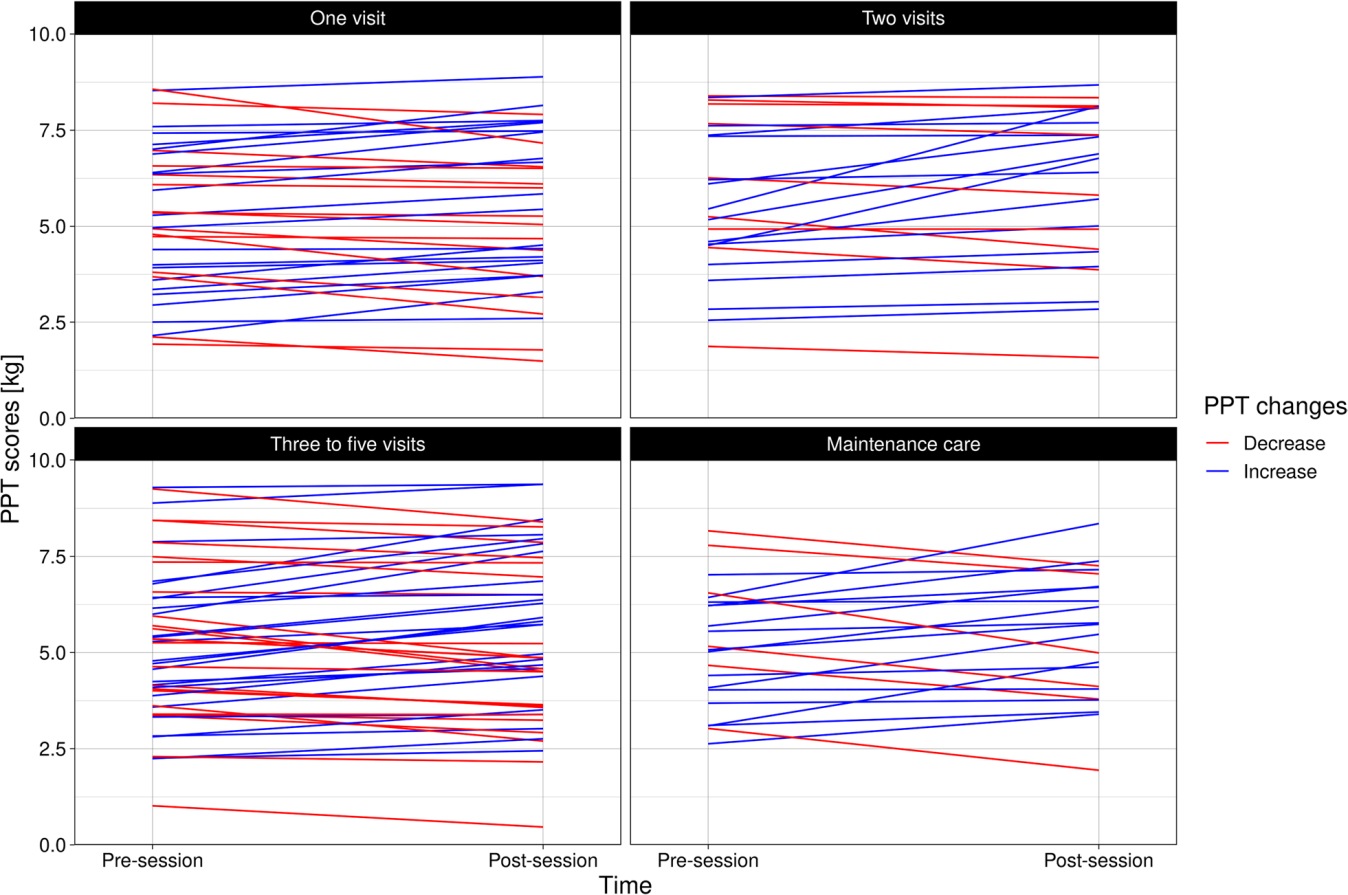
Changes in Pressure pain threshold values from pre to post-session. PPT = Pressure pain threshold

Post-hoc, we considered whether the number of prior visits/treatments was relevant. Most other PPT and SMT research has been conducted with patients or volunteers as discrete treatment sessions rather than structured courses of repeat treatment. Often, the inclusion criteria of such studies have specifically stipulated no SMT treatment in the preceding weeks or months [4–7, 34]. Therefore, we investigated whether a relationship existed between changes in PPT with treatment and the number of preceding visits. We sub-grouped the visits into “first,” “second,” “third-to-fifth,” and “maintenance care” for ease of interpretation. However, this did not provide a visual explanation for our other observations (Fig. 6).

### Aim B—topographic mapping

**Fig. 7:**
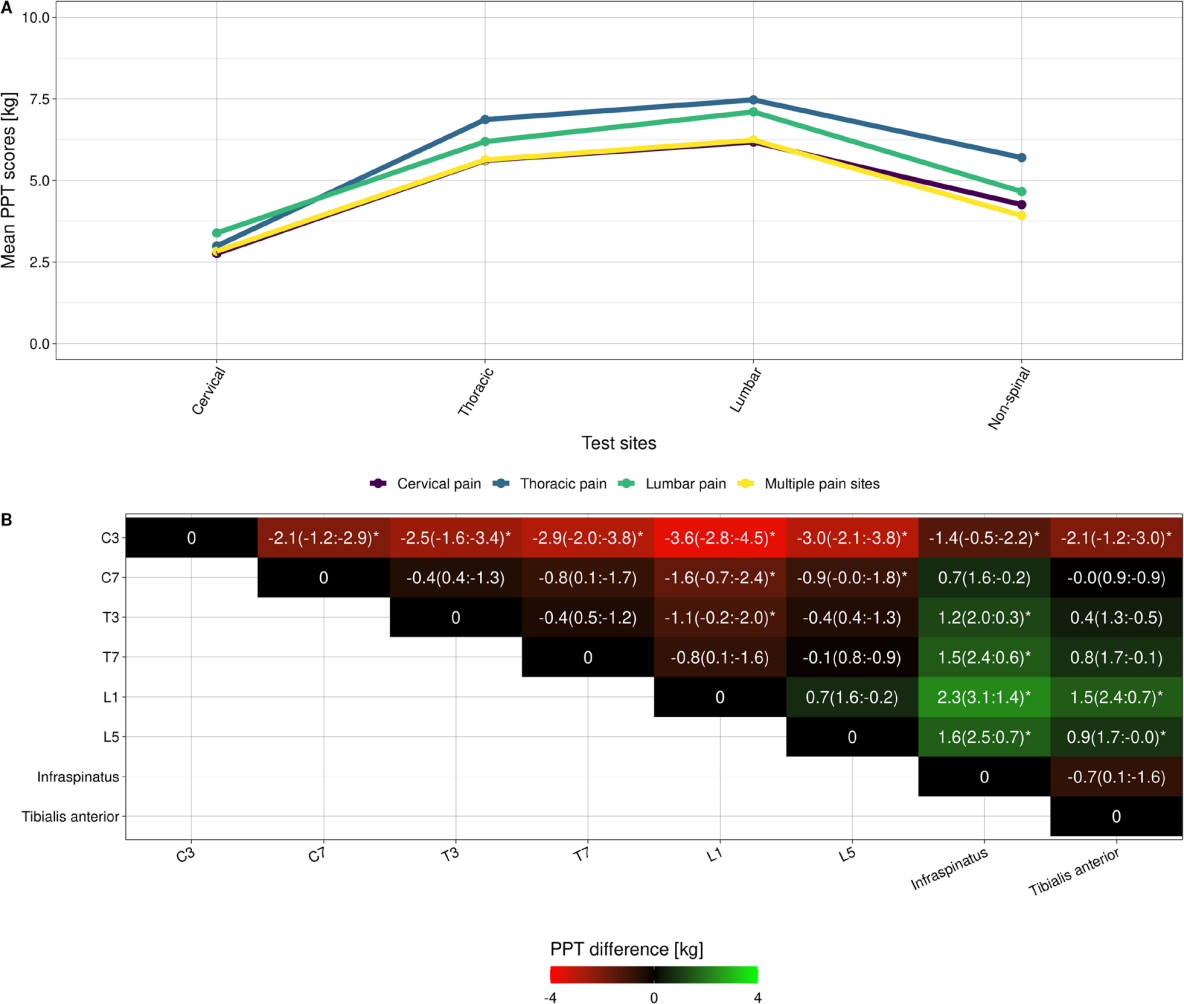
The mean pressure pain threshold values in the region of pain (**A**). Mean PPT difference between test-sites (95% confidence intervals) (**B**). * = *p* value < 0.05, PPT = Pressure pain threshold

The group-mean differences in PPT (pre-session) across test sites were statistically significant when comparing the upper cervical site to any other site and C7 to any lumbar site (Fig. 7A). Generally, the infraspinatus test site had lower PPT values than any other site, except for the cervical test sites. No other consistent findings were observed. The lowest spinal PPT values were in the cervical region and increased caudally (Fig. 7B). There were no differences in the PPT values based on the different clinical pain regions, including those with multiple pain sites compared to participants with pain in a single region. A detailed overview of the summary values per test site can be found at Additional file 5.

### Post-hoc analysis

We attempted to limit intra-individual variation by providing three PPT practice attempts at non-spinal regions before moving to the test sites. However, each site was only tested once. To assess whether a trend was noticeable in the order of PPT testing, we plotted the PPT value at pre-session as a function of the order of test sites. As we limited the pressure to 5 kg on C3, we had to omit this test site. We did not find any trend, and the best-fitting line was close to horizontal across test sites (Beta coefficient = − 0.02 kg, 95%CI = − 0.10 to 0.06 kg) (Additional file 6). This is consistent with our prior topographic study [23].

## Discussion

### Summary of results

This study aimed to investigate immediate changes in PPT following a clinical chiropractic consultation that included SMT provided on the basis of clinical findings. In contrast to prior research, this study was conducted in a real-world clinical setup, with *actual* patients already scheduled for a chiropractic consultation. We assumed that most participants would receive SMT during their consultation [35], and in fact, all participants did, often at several vertebrae, not necessarily limited to their region of pain. This allowed us to investigate changes in PPT in relation to several a-priori selected factors previously shown to affect PPT changes following SMT [8, 9, 36].

Although PPTs were observed to change substantially for some patients, the mean group difference was minimal, and the changes were not associated with any of our a-priori or post-hoc selected independent factors. Overall, immediate changes in PPT did appear not to be related to clinical outcomes in this population of Danish chiropractic patients. Subgroups may exist in relation to changes in PPT after SMT, but if so, they do not seem to be characterized by the clinical response to chiropractic care. While we did see a slightly higher mean increase of PPT at the SMT site, the association between the proximity of the PPT test site and the SMT site was minimal, and noteworthy increases were also observed multiple vertebrae away from the test site. However, we did see a small increase in PPT in those who received myofascial treatment in addition to SMT.

### Spinal manipulation and changes in pressure pain thresholds

These findings do not align with previous publications as we expected to find a systematic increase in PPT independent of all other factors [4–7, 34]. One explanation could be that important differences between a clinical encounter and an experimental setup influence PPT changes following SMT. This raises questions about the generalizability of much previous laboratory-based work in this area.

The clinical encounter and therapeutic alliance between patients and chiropractors, including the trust and reassurance it entails, might influence pain sensitivity [17, 37]. However, a post-hoc analysis of the current data did not indicate any difference in pain sensitivity between new patients and patients attending follow-up visits before or after treatment. Arguably, trust and the therapeutic alliance are built and strengthened over time. Pain testing in a laboratory setup is a very different experience from a clinical encounter, whether as a new patient or a repeat visit, and this could potentially explain why changes are observed with treatment in the laboratory but not in the clinic.

In addition to the different settings (laboratory versus real-world), other factors may explain the lack of a significant PPT increase observed in this study. In QST research, it is well known that the initial pain test often introduces more variance in pain sensitivity than subsequent tests [38–40]. This is likely due to an initial degree of apprehension about unfamiliar painful tests on behalf of study participants. If that is not taken into account, a significant increase in PPT will likely be erroneously attributed to an intervention when in fact, it stems from such non-specific effects. Our study considered this aspect by providing three practice PPT attempts. In a recent randomized controlled trial employing a convincing SMT sham procedure, such considerations were indeed taken into account and employed several practice PPT tests to familiarize participants with the procedure before assessing pre-SMT pain thresholds. The authors found no effect of SMT on PPT measured up to 30 min after treatment [10], and our results echo that conclusion. However, the addition of myofascial treatment may result in small increases in PPT compared to only receiving SMT. While an interplay between these two treatments is anecdotally reported in chiropractic practice, the synergetic effect on SMT-related PPT changes is unknown. PPT changes following myofascial treatment have previously been shown [41], supporting the possibility of an additive effect on PPT when SMT and myofascial techniques are used together.

### Topographic mapping

Our results provide further evidence in the field of topographic mapping in spine pain patients. While our approach was more straightforward than a previous study conducted at our laboratory [23], the results were similar in that PPT values increased the more caudally on the spine we tested. However, there was a negligible difference between adjacent or nearby vertebrae, similar to what we demonstrated earlier in persistent low back pain patients [42]. Also, the region of clinical pain had minimal impact on pre-test PPT, and while the participants with multiple pain sites had the lowest PPT values, it did not reach statistical significance.

### Methodological considerations

While we considered the clinical design an overall strength, especially in contrast to the large body of experimental laboratory research, it could be argued that a clinical design comes at the cost of internal validity [43]. Each clinical encounter is unique by its very nature [44], and each participant will have received individualized treatment, adding variability to the data. Obviously, individual study participants are unique, whether enrolled in a clinical or laboratory study. However, in the present study, we included a mixed population of different pain regions, pain duration, the number of previous visits, etc. Our inclusion criteria were broad, and the participants are likely also heterogeneous on other important aspects such as their psychological state (e.g., mood and anxiety) [45, 46].

We considered our study to reflect real-world chiropractic practice, but we have to recognize that our QST procedure did follow the same stringent prescriptive procedures as other QST studies. However, our study differs in that the SMT was applied pragmatically in an actual clinical setting on patients during the normal course of their care, not volunteer “pain” patients or healthy individuals. Therefore, despite the prescriptive nature of the QST procedures, the study did allow for whatever impact the clinical context may have had on PPT, although we did not quantify these factors. Additionally, using this design, we cannot estimate whether a patient’s experience or knowledge of being involved in a research study impacted the results.

Due to time constraints and multiple testing sites, we limited the PPT assessment to one test per test site. Potentially, our study would have higher reproducibility if two or three trials were used instead. The first PPT trial is likely different from the second, but it is not clear in what direction [38–40]. Some studies report it as higher, while others as lower. We attempted to limit this by using multiple practice attempts. Further, in a post-hoc assessment, we could not find any trend suggesting that the PPT values go in one direction depending on the test sequence. The results of the reliability studies are challenging to translate to our study, as they were all completed on healthy participants. Even if we accept a variance error in our PPT estimates, it would be a systematic error, thereby not impacting our research aims. Also, due to time constraints, we only asked clinicians to report on whether non-SMT treatments were provided but not where. Perhaps myofascial therapy directly at a point related to the PPT test site would result in higher PPT increases, which we cannot explore with our data.

We attempted to assess PPT immediately following their consultation, but we do not know the time lapse between the final SMT and PPT data collection. We attempted to limit this by re-measuring participants (post-session) as soon as possible. The majority of participants received additional care before and/or after SMT, which may have included a combination of other manual treatments, rehabilitation and education, potentially prolonging the time delay between SMT and PPT measurement. The implications of this are unknown as it is uncertain how long a change in PPT following SMT, if present, would last [47]. However, we are undoubtedly not measuring PPT in the refractory SMT period.

We did not use a commercial algometer to capture the PPT, like Somedic or Wagner products. However, the equipment has undergone rigorous validation at our laboratory, with results to be published soon. Further, we have previously published studies using the same type of algometer [24]. We opted to use a spherical probe instead of a circular flat probe to ensure deep-tissue stimulation as opposed to skin irritation theoretically. However, this limits our ability to compare our results with prior publications directly.

### Implications for future research

Considering the existing body of literature, contrasted with our findings and those of a previous methodologically rigorous randomized trial [10], we find that the effect of SMT on pain sensitivity remains unclear. There may be important differences between experimental and clinical settings that impact PPT and the extrapolation of experimental data to clinical settings. In order to move this area forward, more studies are needed which take into account potential sources of error, such as those discussed in the preceding discussion. Also, we have a limited understanding of PPT as a clinically relevant outcome measure—it is not evident that an increase in PPT should be correlated with a clinical improvement [9, 48]. Researchers and clinicians should be cautious about assuming such a relationship exists.

## Conclusion

This clinical study of real-world chiropractic patients failed to find evidence for a substantial generalized increase in mean PPTs following the clinical encounter, including SMT. The PPT did increase substantially for some patients, but no subgroups could be identified associated with substantial increases. The QST results from experimental laboratory setups should not be carelessly extrapolated to clinical settings.

## Supplementary Information

**Additional file 1.** The clinician chart.

**Additional file 2.** Difference in pressure pain threshold from pre-session to post-session for each vertebra.

**Additional file 3.** Difference in pressure pain threshold from pre-session to post-session by self-reported assessment of rapid improvement.

**Additional file 4.** Difference in pressure pain threshold from pre-session to post-session dependent on whether other treatments than SMT were provided.

**Additional file 5.** Pressure pain thresholds at different regions.

**Additional file 6.** PPT scores as a function of the test order.

## Data Availability

Data is available upon reasonable request. Please contact casper.nim@rsyd.dk.

## Abbreviations

QST: Quantitative sensory tests
SMT: Spinal manipulative therapy
HVLA: High-velocity low-amplitude
PPT: Pressure pain threshold
CI: Confidence interval
