# Supplementary material for: Pressure pain thresholds in a real-world chiropractic setting: topography, changes after treatment, and clinical relevance?
Source: Chiropr Man Therap. 2022 May 12;30:25. doi: 10.1186/s12998-022-00436-2 (PMC9097359; doi:10.1186/s12998-022-00436-2)

Supplementary material 1

## The clinician chart

The clinician chart used to extract clinician-reported variables.


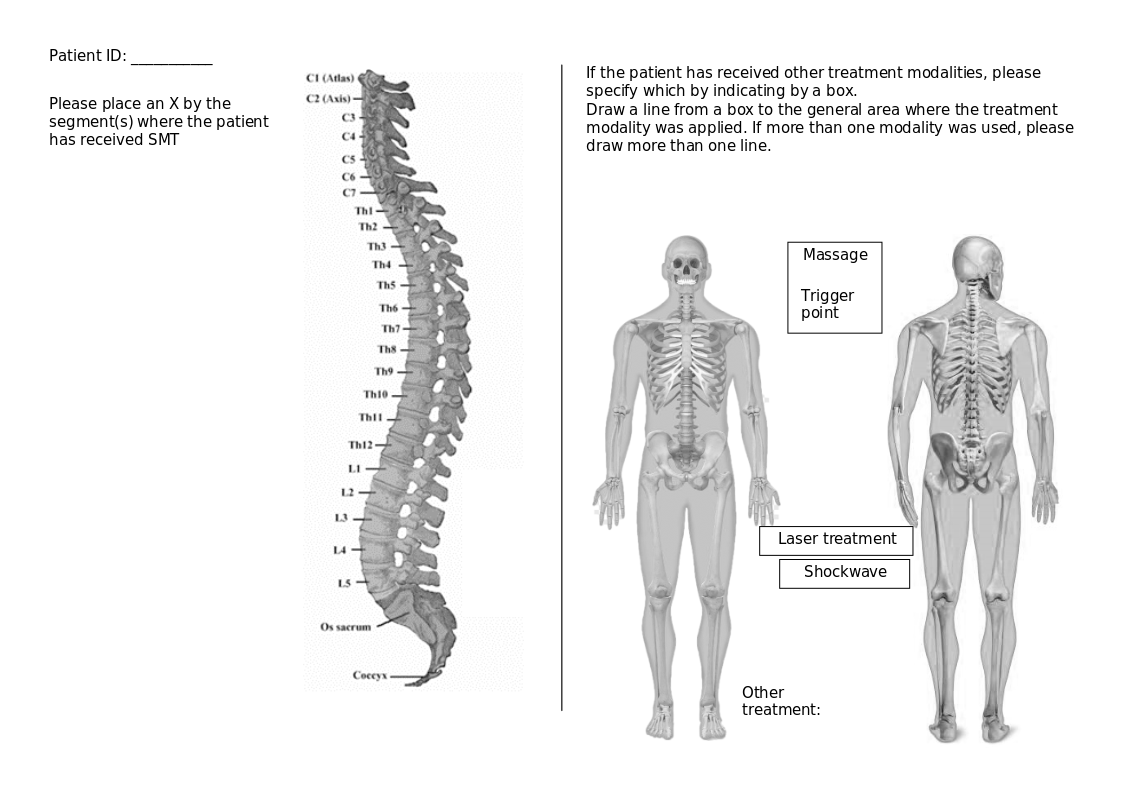

Supplement: Supplementary file 1 — Additional file 1. The clinician chart. [file 12998_2022_436_MOESM1_ESM.docx]
