# Supplementary material for: Pressure pain thresholds in a real-world chiropractic setting: topography, changes after treatment, and clinical relevance?
Source: Chiropr Man Therap. 2022 May 12;30:25. doi: 10.1186/s12998-022-00436-2 (PMC9097359; doi:10.1186/s12998-022-00436-2)
Supplement: Supplementary file 2 — Additional file 2. Difference in pressure pain threshold from pre-session to post-session for each vertebra. [file 12998_2022_436_MOESM2_ESM.docx]

Supplementary material 2

## Difference in pressure pain threshold from pre-session to post-session for each vertebra

Difference in pressure pain threshold from pre-session to post-session for each vertebra in Danish chiropractic patients.

| Test site | Mean.change | CI_low | CI_high | P-value |
| --- | --- | --- | --- | --- |
| C3 | -0.15 | -0.73 | 0.42 | 1.00 |
| C7 | -0.20 | -0.77 | 0.37 | 1.00 |
| T3 | -0.16 | -0.73 | 0.41 | 1.00 |
| T7 | -0.33 | -0.90 | 0.24 | 0.80 |
| L1 | -0.20 | -0.77 | 0.38 | 1.00 |
| L5 | -0.49 | -1.06 | 0.09 | 0.17 |
| Infraspinatus | -0.16 | -0.73 | 0.41 | 1.00 |
| Tibialis Ant. | 0.10 | -0.47 | 0.67 | 1.00 |
| N = 129, PPT mean changes from pre to post consultation (95% CI) for each segment | | | | |

## 
