# Supplementary material for: Pressure pain thresholds in a real-world chiropractic setting: topography, changes after treatment, and clinical relevance?
Source: Chiropr Man Therap. 2022 May 12;30:25. doi: 10.1186/s12998-022-00436-2 (PMC9097359; doi:10.1186/s12998-022-00436-2)
Supplement: Supplementary file 3 — Additional file 3. Difference in pressure pain threshold from pre-session to post-session by self-reported assessment of rapid improvement. [file 12998_2022_436_MOESM3_ESM.docx]

Supplementary material 3

## Difference in pressure pain threshold from pre-session to post-session by self-reported assessment of rapid improvement

Difference in pressure pain threshold from pre-session to post-session dependent on Danish chiropractic patients self-reported assessment of rapid improvement.

| Subjective change (Difference between responders) | PPT Difference from pre to post consultation (95% CI) |
| --- | --- |
| No.change - Better | 0.00 (-0.55-0.56) |
| No.change - Little.better | 0.07 (-0.49-0.62) |
| No.change - Much.better | 0.17 (-0.48-0.82) |
| Little.worse - Better | 0.49 (-0.49-1.46) |
| Little.worse - Little.better | 0.55 (-0.43-1.53) |
| Little.worse - Much.better | 0.65 (-0.38-1.69) |
| Little.worse - No.change | 0.48 (-0.52-1.48) |
| Little.better - Better | -0.06 (-0.58-0.45) |
| Little.better - Much.better | 0.10 (-0.51-0.72) |
| Better - Much.better | 0.17 (-0.45-0.78) |
| N = 129 | |

## 
