# Supplementary material for: Pressure pain thresholds in a real-world chiropractic setting: topography, changes after treatment, and clinical relevance?
Source: Chiropr Man Therap. 2022 May 12;30:25. doi: 10.1186/s12998-022-00436-2 (PMC9097359; doi:10.1186/s12998-022-00436-2)
Supplement: Supplementary file 4 — Additional file 4. Difference in pressure pain threshold from pre-session to post-session dependent on whether other treatments than SMT were provided. [file 12998_2022_436_MOESM4_ESM.docx]

Supplementary material 4

## Difference in pressure pain threshold from pre-session to post-session dependent on whether other treatments than SMT were provided

Difference in pressure pain threshold from pre-session to post-session dependent on whether other treatments than SMT were provided for Danish chiropractic patients.

| Parameter | Difference in change in PPT between different non-SMT treatments (95% CI) |
| --- | --- |
| Massage to Multiple | -0.14 (-1.26-0.97) |
| Massage to Muscle energy technique | -0.61 (-2.28-1.05) |
| Massage to None | 0.20 (-0.39-0.79) |
| Massage to Other | -0.08 (-2.38-2.21) |
| Multiple to None | 0.34 (-0.72-1.40) |
| Multiple to Other | 0.06 (-2.40-2.52) |
| Muscle energy technique to Multiple | 0.47 (-1.41-2.35) |
| Muscle energy technique to None | 0.81 (-0.81-2.44) |
| Muscle energy technique to Other | 0.53 (-2.22-3.28) |
| Myofascial to Massage | 0.33 (-0.24-0.91) |
| Myofascial to Multiple | 0.19 (-0.86-1.24) |
| Myofascial to Muscle energy technique | -0.28 (-1.90-1.34) |
| Myofascial to None | 0.53 (0.08-0.98)* |
| Myofascial to Other | 0.25 (-2.02-2.52) |
| Other to None | 0.28 (-1.99-2.55) |
| N = 129 | |

*Myofasciel includes compression techniques and dryneedling. Other includes laser therapy and free text field comments. * = p-value < 0.05*
