# Supplementary material for: Pressure pain thresholds in a real-world chiropractic setting: topography, changes after treatment, and clinical relevance?
Source: Chiropr Man Therap. 2022 May 12;30:25. doi: 10.1186/s12998-022-00436-2 (PMC9097359; doi:10.1186/s12998-022-00436-2)
Supplement: Supplementary file 5 — Additional file 5. Pressure pain thresholds at different regions. [file 12998_2022_436_MOESM5_ESM.docx]

Supplementary material 5

## Pressure pain thresholds at different regions

Summary statistics of pressure pain thresholds at different regions for Danish chiropractic patients.

| Vertebra | Mean | Median | Standard deviation | Interquartile range |
| --- | --- | --- | --- | --- |
| C3 | 3.11 | 3.12 | 1.25 | 2.04 |
| C7 | 5.18 | 4.90 | 2.31 | 3.39 |
| T3 | 5.63 | 5.06 | 2.48 | 3.80 |
| T7 | 5.99 | 5.77 | 2.25 | 3.35 |
| L1 | 6.74 | 6.66 | 2.49 | 4.27 |
| L5 | 6.06 | 6.11 | 2.57 | 3.78 |
| Infraspinatus | 4.47 | 4.24 | 2.07 | 2.73 |
| Tibialis anterior | 5.21 | 5.02 | 2.30 | 3.29 |
| N = 129 | | | | |

## 
