# Supplementary material for: Pressure pain thresholds in a real-world chiropractic setting: topography, changes after treatment, and clinical relevance?
Source: Chiropr Man Therap. 2022 May 12;30:25. doi: 10.1186/s12998-022-00436-2 (PMC9097359; doi:10.1186/s12998-022-00436-2)

Supplementary material 6

## PPT scores as a function of the test order

A jitter plot showing the best-fitting line of PPT at pre-session as a function of the order of test sites.


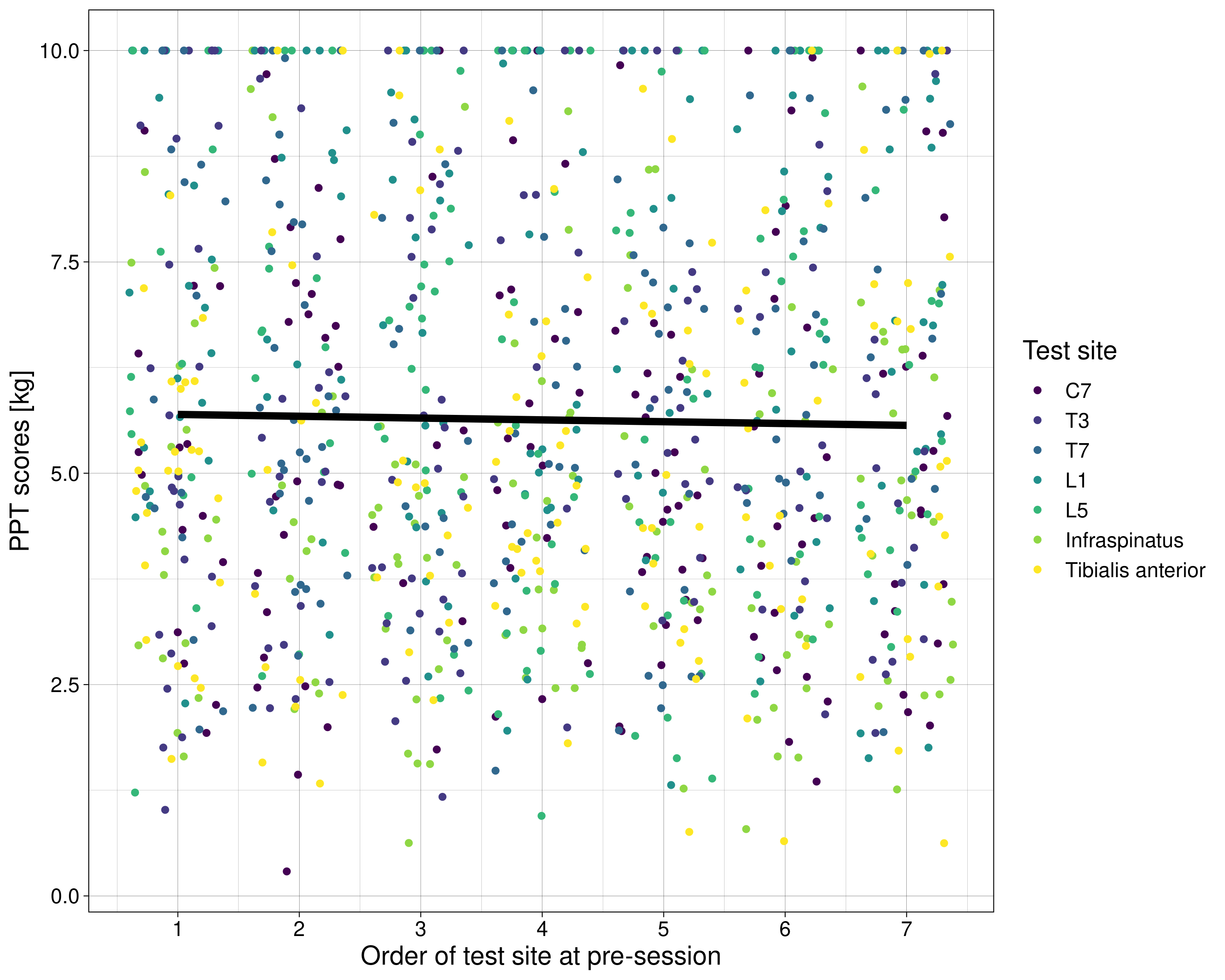

Supplement: Supplementary file 6 — Additional file 6. PPT scores as a function of the test order. [file 12998_2022_436_MOESM6_ESM.docx]
